# Senescence‐associated transcriptional derepression in subtelomeres is determined in a chromosome‐end‐specific manner

**DOI:** 10.1111/acel.13804

**Published:** 2023-03-15

**Authors:** Martin Rey‐Millet, Mélanie Pousse, Chan Soithong, Jing Ye, Aaron Mendez‐Bermudez, Eric Gilson

**Affiliations:** ^1^ CNRS, INSERM, IRCAN, Faculty of Medicine Nice Université Côte d'Azur Nice France; ^2^ Department of Geriatrics, Medical center on Aging of Shanghai Ruijin Hospital Shanghai Jiaotong University School of Medicine Shanghai China; ^3^ International Laboratory in Hematology, Cancer and Aging, Pôle Sino‐Français de Recherches en Sciences du Vivant et Génomique, RuiJin Hospital Shanghai Jiao Tong University School of Medicine/CNRS/INSERM/University Côte d’Azur Shanghai China; ^4^ Department of medical genetics CHU Nice France

**Keywords:** polycomb complex, replicative senescence, subtelomere, telomeres, telomere position effect

## Abstract

Aging is a continuous process leading to physiological deterioration with age. One of the factors contributing to aging is telomere shortening, causing alterations in the protein protective complex named shelterin and replicative senescence. Here, we address the question of the link between this telomere shortening and the transcriptional changes occurring in senescent cells. We found that in replicative senescent cells, the genes whose expression escaped repression are enriched in subtelomeres. The shelterin protein TRF2 and the nuclear lamina factor Lamin B1, both downregulated in senescent cells, are involved in the regulation of some but not all of these subtelomeric genes, suggesting complex mechanisms of transcriptional regulation. Indeed, the subtelomeres containing these derepressed genes are enriched in factors of polycomb repression (EZH2 and H3K27me3), insulation (CTCF and MAZ), and cohesion (RAD21 and SMC3) while being associated with the open A‐type chromatin compartment. These findings unveil that the subtelomere transcriptome associated with senescence is determined in a chromosome‐end‐specific manner according to the type of higher‐order chromatin structure.

## Graphical abstract

Using human primary fibroblasts, here we report enrichment in a subset of human subtelomeres of genes embedded into polycomb heterochromatin (H3K27me3) and upregulated in senescent cells via an attenuation of polycomb repression.

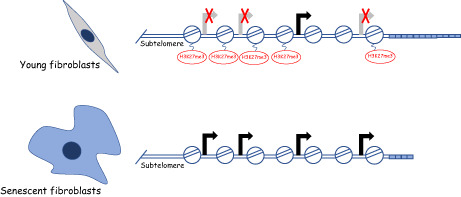

**Abbreviations**
DDR: DNA damage response
DEGs: differentially expressed genesx
IRIS: ionizing radiation‐induced senescence
OIS: oncogene induced‐senescence
PD: population doubling
RS: replicative senescence
TPE: telomere position effect

## 1 INTRODUCTION

The biology of aging studies the mechanisms leading to a functional decline as a function of age, whether at the molecular, cellular, tissue, or systemic level (Gorgoulis et al., 2019; López‐Otín et al., 2013). Over the past two decades, our understanding of aging biology has made remarkable progress allowing the identification of unifying features (López‐Otín et al., 2013). Among them, the accumulation of senescent cells appears as a major contributor to physiological and pathological aging (Anderson et al., 2019; Baker et al., 2011, 2016). However, the integration and time‐dependent relationship between these aging pathways in different organs and major physiological functions as well as their roles in age‐related diseases remain largely unrecognized (Roy et al., 2020). Various aging clocks have been recently described: transcription (Meyer & Schumacher, 2021), methylation (Horvath, 2013), and inflammation (Sayed, 2021), providing interesting tools to assay for biological age but we are still facing a “cause or consequence” challenge. Thus, there is a need to decipher the time‐dependent and causality relationship existing between the different aging pathways.

The ends of chromosomes are protected from unwanted DNA damage response (DDR) by the shelterin protein complex that specifically binds to telomeric DNA. In humans, the shelterin is composed of six proteins: TRF1 and TRF2, binding the double‐stranded DNA part of telomeres; POT1, binding the 3′‐overhang; RAP1, binding to TRF2; and TIN2/TPP1, forming a protein bridge between TRF1/TRF2 and POT1 (de Lange, 2018; Ghilain et al., 2021). In addition, telomeric chromatin is associated with the non‐coding RNA TERRA transcribed from subtelomeric regions and can adopt peculiar conformations involving telomeric DNA looping (T‐loop; Griffith et al., 1999). The formation of T‐loops, which is facilitated by the shelterin subunit TRF2, contributes to the prevention of DDR checkpoint and repair activation (Benarroch‐Popivker et al., 2016; Doksani et al., 2013; Sarek et al., 2019). The replication of telomeric DNA requires specific mechanisms to compensate for the inherent inability of the replication machinery to fully duplicate the extremities of the parental DNA molecule (Gilson & Géli, 2007). In many organisms, this involves a specialized reverse transcriptase, the telomerase (Blackburn et al., 2006).

Telomere shortening is programmed during normal development in humans and other vertebrate species. Indeed, in these organisms, the telomerase expression is downregulated at the end of embryogenesis in somatic tissues leading to telomere shortening at each cell division, eventually resulting in replicative senescence when a subset of telomeres becomes too short to ensure their anti‐checkpoint functions (Hayflick limit; Abdallah et al., 2009; Bodnar et al., 1998; Kaul et al., 2011). Throughout life, the rate of telomere shortening is paced as a function of the regeneration properties of the considered cell (Demanelis et al., 2020) and in response to external stressors (Jacome Burbano & Gilson, 2021). Telomere shortening can be a driver of human aging since germinal mutations of the telomerase and shelterin complexes, leading to critical telomere shortening and deprotection, cause rare progeroid syndromes such as Dyskeratosis Congenita (Armanios & Blackburn, 2012). Moreover, short telomeres are associated in the general population with a broad spectrum of age‐related pathologies, including cardiovascular and neurodegenerative diseases, type II diabetes, pulmonary fibrosis, and arthrosis (Blackburn et al., 2015; Martínez & Blasco, 2017).

A function of telomeres is to modulate gene expression. This was first discovered in yeast for the process leading to the repression of subtelomeric genes, named telomere position effect (TPE; Gottschling et al., 1990). In brief, in this organism, the major shelterin subunit, Rap1, serves to recruit a heterochromatin complex at telomeres, including the sirtuin Sir2, followed by its spreading toward the centromere (Kueng et al., 2013). The subtelomeric genes succumbing to this TPE silencing are determined in a chromosome‐end‐specific manner by specific combinations of insulators and proto‐silencer sequences, leading to subtelomeric chromatin loops that regulate the heterochromatin spreading in a discontinuous manner (Fourel et al., 1999; Lebrun et al., 2003; Miele et al., 2009). Interestingly, the yeast subtelomeric genes whose expression is regulated by telomeres are related to stress responses and could thus contribute to an adaptive response in case of telomere dysfunction (Ai et al., 2002; Platt et al., 2013). Since these pioneer studies, the existence of TPE was reported in various organisms, including humans (Baur et al., 2001; Koering et al., 2002). Our understanding of the molecular mechanisms of human TPE remains fragmented with the involvement of TERRA (Arnoult et al., 2012), a shelterin subunit (TRF2) (Deng et al., 2009; Kim et al., 2016; Robin et al., 2014), a sirtuin enzyme (SirT6; Tennen et al., 2011), and long‐range chromatin loops within the subtelomeric region (Robin et al., 2020; Wood et al., 2014). In particular, the heterochromatin nature of human telomeres remains largely indetermined since in many cell types the telomeres do not exhibit classical heterochromatin marks (Cubiles et al., 2018; Gauchier et al., 2019). Thus, despite striking similarities with yeast, no comprehensive model is established yet for human TPE. Telomeres can also modulate the expression of genes located in the interior of chromosomes by sequestering transcription factors at telomeres (Gotta et al., 1996; Maillet et al., 1996; Marcand et al., 1996). In the case of telomere shortening, their release throughout the nucleoplasm leads to genome‐wide transcriptional regulation of targeted genes (Platt et al., 2013; Ye et al., 2014).

The link between telomere and gene expression led to the hypothesis that telomere shortening contributes to the transcriptional changes associated with aging (Maillet et al., 1996; Wright & Shay, 1992; Ye et al., 2014). This view was first supported in yeast replicative senescence (Platt et al., 2013) and recently in human aging transcriptomic studies revealing that telomere length correlates with age‐related gene expression (Demanelis et al., 2020) and that genes upregulated with aging are enriched in subtelomeric regions (Dong et al., 2021).

In this work, we analyzed, by RNA sequencing, the transcriptional changes occurring at replicative senescence. We found a significant enrichment of derepressed genes at senescence in regions confined to two megabases from telomeres. Some but not all of them are sensitive to TRF2 and Lamin B1 dosage. Noteworthy, these genes are clustered in a subset of subtelomeres enriched in factors controlling chromatin higher‐order structure such as CTCF, MAZ, and the cohesion complex. They are also enriched in chromatin marks of repression by the polycomb complex (EZH2 and H3K27me3) in contrast to the other subtelomeres rather enriched in marks of repression by HP1 (H3K9me3).

## 2 RESULTS

### 2.1 Subtelomeric gene derepression during replicative senescence is restricted to a subset of subtelomeres

**FIGURE 1:**
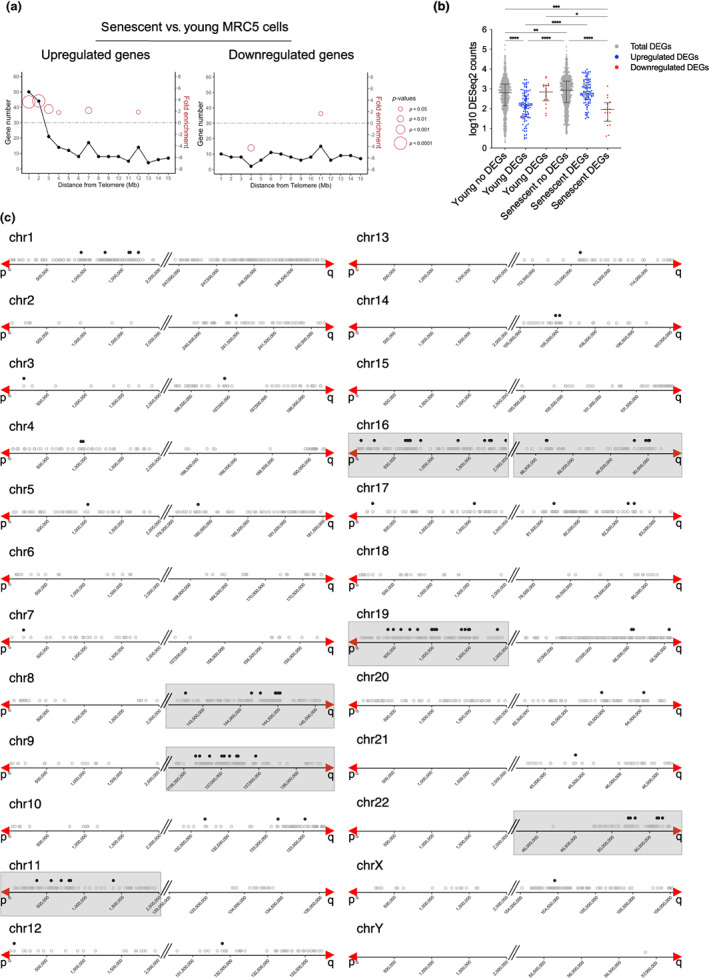
Distribution of DEGs at senescence. (a) Distribution of DEGs in senescent (PD 71) versus young (PD 30) MRC‐5 cells. Upregulated (left) and downregulated (right) genes are shown. The line plots illustrate the number of DEGs related to their distance to telomeres in 1 Mb intervals. Fold enrichment (right *Y*‐axis) is only shown if there is a significant *p*‐value and the size of the circles corresponds to the *p*‐value of the enrichment obtained by a hypergeometric test. (b) Dot plot of DESeq2 normalized counts from the first 2 Mb of telomeres. Counts from non‐differentially expressed genes (no DEGs) and from DEGs in the two conditions (young and senescent) are plotted. Upregulated DEG counts are plotted in blue and downregulated DEG counts are plotted in red. Data represent median with interquartile range of three biological replicates. Statistical analyses were performed using the Kruskal–Wallis test, (*p < 0.05; **p < 0.001; ***p < 0.0001; ****p < 0.00001). (c) Upregulated genes in the first 2 Mb from telomeres. The grey empty dots represent all protein‐coding genes and pseudogenes. The black dots represent upregulated genes (senescent versus young MRC‐5 cells) from this work. The red arrows mark the start of the telomere. The grey boxes delimitate the subtelomeres that are significantly enriched in upregulated genes.

To investigate the transcriptional regulation occurring at replicative senescence (RS), we cultured human lung primary fibroblasts (MRC‐5 cells) at 5% oxygen until they senesce (population doubling, PD, 71). We defined a senescent culture when cells stop proliferating and are positive for senescence‐associated *β*‐galactosidase (SA‐*β*‐Gal) staining, and EdU is incorporated in <1% of the cells (Figure S1A,B). We harvested young (PD 30) and RS (PD 71) MRC‐5 cells in triplicates, followed by RNA sequencing (RNA‐seq) and differential expression analysis. This allowed us to identify 1051 differentially expressed genes (DEGs). When plotted according to the distance to telomeres, the upregulated DEGs found at the first 2 Mb from the telomeres were positively enriched (fold enrichment = 3.6 and 3.8 with *p* = 7.86 e^−15^ and 7.13 e^−14^, respectively, hypergeometric test, Figure 1a). On the contrary, no specific enrichment was observed in subtelomeric regions for the downregulated DEGs (Figure 1a).

We hypothesized that the DEG upregulation could result from a senescence‐associated process of either derepression or specific transactivation. To decipher between these two possibilities, we compared the expression levels of the DEGs and no‐DEGs in young and senescent cells by analyzing the RNA‐seq counts. We used the DESeq2 normalized counts of genes located in the first 2 Mb from telomeres, and we separated them into upregulated and downregulated genes (Figure 1b). As expected, this analysis revealed a significant increase in read counts in senescent versus young MRC‐5 of the upregulated DEG group (*p* < 0.0001, Kruskal–Wallis test, Figure 1b). Notably, there were significantly fewer reads in the young upregulated DEG group as compared to the no‐DEG group (*p* < 0.0001, Kruskal–Wallis test), indicating that the upregulated DEGs are repressed in young cells compared to the no‐DEGs where no change is evident. Therefore, since the upregulated DEGs are less expressed in young cells than the no‐DEGs, their upregulation in senescent cells is better explained by a senescence‐associated mechanism of derepression.

Regarding downregulated DEGs (Figure 1b), the read counts in the senescent DEG group were significantly lower compared to the young DEG group (*p* = 0.0151, Kruskal–Wallis test). This indicates that downregulated DEGs in senescent cells, at least in subtelomeric regions, result from senescence‐associated transcriptional repression.

Next, we investigated whether the increase of subtelomeric transcription at senescence is a global feature of subtelomeres. We looked for positive fold enrichment of upregulated genes chromosome by chromosome by defining bins of 2 Mb. The enrichment was calculated considering gene density (represented by the grey dots, Figure 1c; see the method section for further details). We found seven chromosome ends showing significant positive enrichment of upregulated genes at the first 2 Mb from the telomeres (chromosome arms 8q, 9q, 11p, 16p, 16q, 19p, and 22q, Figure 1c). On the contrary, only one chromosome arm (5p) was enriched for downregulated genes (Figure S2).

### 2.2 The shelterin protein TRF2 contributes to the senescence‐associated subtelomeric gene expression

We asked whether gene expression in subtelomeres can be regulated by the shelterin protein TRF2, which is downregulated as cells approach senescence (Fujita et al., 2010; Mendez‐Bermudez et al., 2022) and involved in subtelomeric gene expression (Robin et al., 2014, 2020).

**FIGURE 2:**
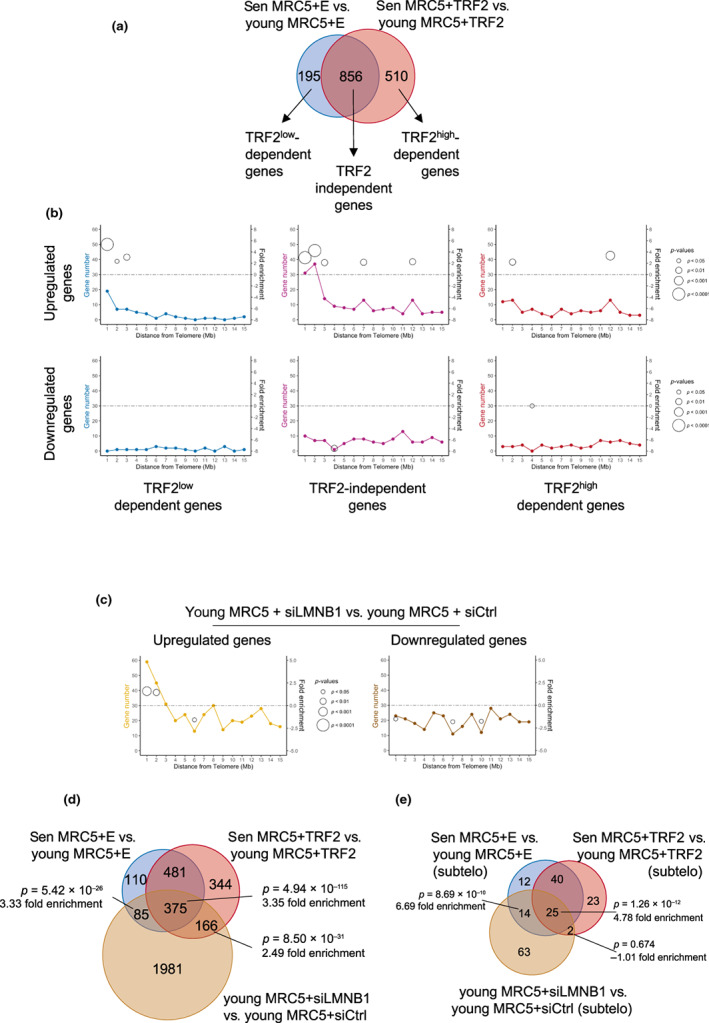
Distribution of DEGs influenced by TRF2 and Lamin B1. (a) Venn diagram of DEGs found in senescent (sen) versus young MRC‐5 cells infected with an empty (E, blue) or a TRF2 expressing lentivirus vector (TRF2, red) respectively. Cells transduced with an empty vector senesce at a population doubling (PD) of 71, while cells expressing TRF2 at a PD of 75. Young cells were transduced at PD 30. (b) Distribution of DEGs influenced by TRF2 in the first 15 Mb from telomeres is shown. Upregulated (upper panel) and downregulated (lower panel) genes for the 3 categories of genes described in a are presented. Fold enrichment is only shown if there is a significant *p*‐value and the size of the circles corresponds to the *p*‐value of the enrichment obtained by a hypergeometric test. (c) Distribution of DEGs in young (PD 30) MRC‐5 cells transfected with siLMNB1 or siControl. The first 15 Mb from the telomere are shown. The line charts illustrate the number of DEGs related to their distance to telomeres in 1 Mb bins. Significant fold enrichment (right *Y*‐axis) is marked with open circles. The *p*‐value of the enrichment was obtained by a hypergeometric test. (d) Venn diagram of DEGs found between senescent cells and young MRC‐5 cells transduced with an empty or TRF2 vector (as shown in 2a) and young MRC‐5 cells with downregulation of Lamin B1 (brown circle). Fold enrichment was calculated, and the corresponding *p*‐value was estimated using a hypergeometric test. (e) Similar comparison as in D but only upregulated genes present at the first 2 Mb from the telomeres were used for the analysis. Fold enrichment and *p*‐value were estimated using a hypergeometric test.

To this end, we infected pre‐senescent MRC‐5 cells with a TRF2 expressing lentivirus or an empty control and extracted RNA when the cells reached senescence. In the case of the TRF2‐expressing cells, senescence was reached after 75 PD in culture, while the control cells transduced with an empty vector stopped dividing at PD 71. In parallel, we ectopically expressed TRF2 in young MRC‐5 cells (PD 30) for 6 days before RNA extraction. In both, young and RS cells, the levels of TRF2 were high but constant (Figure S3A). After RNA sequencing and differential expression analysis, we identified 1366 DEGs when we compared senescent vs young MRC‐5 over‐expressing TRF2 and 1051 DEGs in senescent versus young MRC‐5 transduced with an empty vector (Figure 2a). By crossing the two datasets, we obtained three groups of DEGs (Figure 2a) which are dependent upon senescence but that behaved differently according to the ectopic expression of TRF2. The intersection between the two datasets identified 856 genes that are not influenced by the TRF2 levels. We called them TRF2‐independent DEGs. We found 510 DEGs detected only when TRF2 is overexpressed; thus, we named this group as TRF2^high^‐dependent DEGs. Finally, we found 195 DEGs detected only in the empty control group. We assumed that the expression regulation of this group of genes is influenced by the natural decrease of the TRF2 protein level seen in senescent cells and we named this group TRF2^low^‐dependent DEGs. We confirmed the results obtained from our RNA‐seq for some of these DEGs by RT‐qPCR (Figure S3B).

Similar to what we found for the whole transcriptome of senescent cells (Figure 1a), enrichment at subtelomeres was found for the upregulated TRF2‐independent DEGs (fold enrichment = 3.0 and 4.3 with *p* = 7.57 e^−8^ and 1.83 e^−13^, hypergeometric test) and TRF2^*low*^‐dependent DEGs (fold enrichment = 5.3 and 2.3 with *p* = 2.87 e^−9^ and 0.031, hypergeometric test) but not for the TRF2^high^‐dependent DEGs (Figure 2b). These results suggest that TRF2 downregulation upon telomere shortening is responsible for the derepression of some of the subtelomeric genes. Interestingly, out of the 195 TRF2^low^‐dependent DEGs, 27 are subtelomeric and from those, 26 were upregulated DEGs, showing that the naturally low levels of TRF2 at senescence lead to an upregulation of subtelomeric genes.

Next, we asked whether TRF2 levels also regulate the expression of the TRF2^low^‐dependent DEGs in a non‐senescent context, i.e., in young cells of the same cell line. Indeed, downregulating TRF2 in young MRC‐5 cells was enough to cause an increased expression of genes belonging to the TRF2^low^‐dependent category (Figure S3C), further demonstrating the role of TRF2 in the replicative senescence transcriptome.

Gene ontology analyses using Reactome reveal that the TRF2‐independent DEGs belong to pathways related to cell cycle arrest and senescence (Figure S3D). The other two groups, TRF2^low^ and TRF2^high^‐dependent DEGs, did not show a particular pathway enrichment probably due to the low number of DEGs present in those groups.

### 2.3 Decreased Lamin B1 expression leads to subtelomeric gene derepression

Lamin B1 is an important regulator of chromatin architecture and a component of the nuclear lamina whose expression decreased at senescence (Freund et al., 2012; Shimi et al., 2011). Together with the evidence of interactions between TRF2 and Lamin B1 (Pennarun et al., 2021), the fact that senescence‐associated TRF2 downregulation reduces the amount of Lamin B1 at heterochromatin (Mendez‐Bermudez et al., 2022) suggests a mechanistic link between senescence, TRF2, Lamin B1 and gene expression regulation. Thus, we performed RNA‐seq with young (PD 30) MRC‐5 cells upon Lamin B1 knock‐down and control (Figure S4A). We identified 2607 DEGs (*p* adjusted < 0.05). Among them, 104 were upregulated subtelomeric genes and enriched in subtelomeric regions (fold enrichment = 1.7 with *p* = 3.01 e^−4^ for the first Mb from the telomere and fold enrichment = 1.5 with *p* = 0.0085 for the second Mb from the telomere, Figure 2c and Figure S4B), but no enrichment was observed for downregulated genes (Figure 2c). These results are similar to replicative senescence (Figure 1a) or upon TRF2 expression (Figure 2b) where no enrichment was found for downregulated genes. Interestingly, the two subtelomeres enriched upon knocking down of Lamin B1 (chr 8q and chr 22q, Figure S4C,D) belong to the group of seven chromosome ends being positively enriched in upregulated DEG at senescence (see Figure 1c).

Among the 2607 Lamin B1‐dependent regulated genes, 460 were also differentially expressed in RS, in agreement with the decreased expression of Lamin B1 at senescence (Figure 2d). Moreover, a large majority of TRF2^low^‐dependent and TRF2‐independent DEGs are significantly overlapping the subtelomeric Lamin B1‐dependent upregulated DEGs (fold enrichment = 6.7 and 4.8 with *p* = 8.69 e^−10^ and *p* = 1.26 e^−12^, respectively, Figure 2e).

We concluded that the Lamin B1 downregulation in young fibroblasts recapitulates in part the transcriptome of senescent cells, including the subtelomere‐specific enrichment of derepressed genes. Noteworthy, the majority of TRF2^low^‐dependent DEGs are also regulated by Lamin B1 in young cells, suggesting commonalities between TRF2 and Lamin B1 to regulate their expression.

### 2.4 Specific subtelomeric gene derepression is not restricted to replicative senescence

**FIGURE 3:**
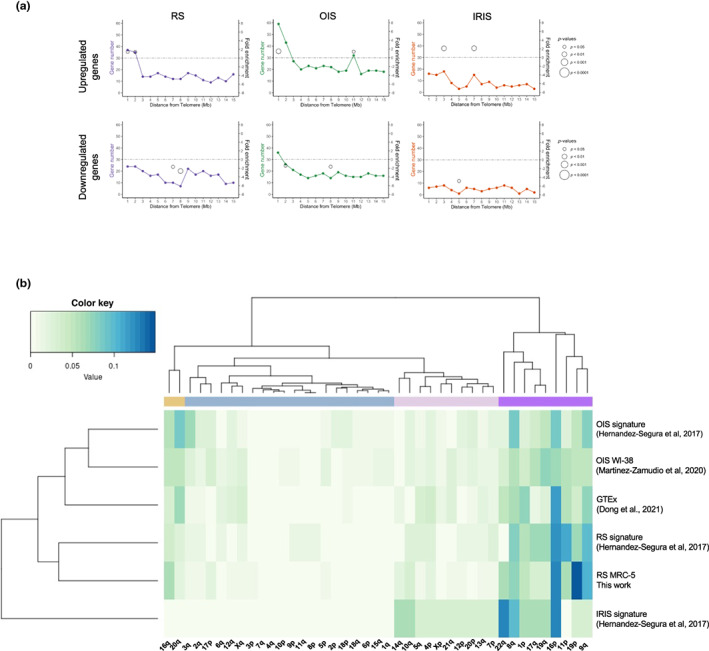
Subtelomeric DEGs distribution of different senescence inducers. (a) Distribution of upregulated genes (top panel) and downregulated genes (bottom panel) specific to a senescence stimulus identified by Hernandez‐Segura et al. (2017), along the first 15 Mb from telomeres (RS, Replicative Senescence; OIS, Oncogene Induced‐Senescence; IRIS, Ionizing Radiation‐Induced Senescence). The plot chart shows the number of senescence‐specific genes from each stimulus in relation to their distance to telomeres. Fold enrichment (right *Y*‐axis) is only shown if there is a significant *p*‐value and the size of the circles corresponds to the *p*‐value of the enrichment obtained by a hypergeometric test. (b) Heat map showing the clustering of upregulated genes in the first 2 Mb from telomeres per chromosome end. The main clusters are depicted with different colors at the top. GTEx, Genotype‐Tissue Expression project; IRIS, Ionizing Radiation‐Induced Senescence; OIS, Oncogene Induced Senescence; RS, Replicative Senescence.

We then asked whether the subtelomeric gene expression profile identified here in the context of “pure” replicative senescence (i.e., cells grown at 5% oxygen to avoid a combined effect with exogenous oxidative stress) is also observed when senescence is induced by other stressors. To this end, we compared our RNA‐seq results with a compilation of RNA‐seq datasets from different cell and senescence types (Hernandez‐Segura et al., 2017), including transcriptional signatures for replicative senescence (RS; comprising BJ, HFF, MRC‐5, WI‐38 and IMR‐90 cells), oncogene‐induced senescence (OIS; IMR‐90) and ionizing radiation‐induced senescence (IRIS; HCA‐2). Similar to our RNA‐seq dataset, when plotted along chromosomes, the RS signature genes but not those of the IRIS one exhibited a subtelomeric enrichment for upregulated genes (fold enrichment = 1.5 and 1.4 with *p* = 0.015 and 0.028 for the first and second Mb from the telomere, Figure 3a). Subtelomeric enrichment of upregulated genes was also detected in the OIS signature group (fold enrichment = 1.5 with *p* = 0.0017; Figure 3a).

We asked which chromosome ends were enriched for upregulated DEGs in these datasets. We found enrichment in three chromosome ends (9q, 11p, and 16p) for the RS signature and five (3q, 8q, 9q, 16q, and 20q) for the OIS signature (Table S1). Interestingly, most of those chromosome ends overlap with the seven chromosome ends described for our RNA‐seq with MRC‐5, although not necessarily the same genes are differentially expressed.

It was previously shown that age‐dependent DEGs in human tissues were enriched in subtelomeres (Dong et al., 2021). Thus, we asked whether the chromosome ends described in this work (MRC‐5 RS cells) were also enriched in age‐dependent DEGs. We found that five out of the seven subtelomeres were enriched for age‐dependent genes (Table S1) but not necessarily for the same genes. This suggests a combined action of subtelomeric‐specific properties and cell‐type‐specific mechanisms acting at the gene level.

By combining the results of our RNA‐seq analysis (MRC‐5 RS) with those of Hernandez‐Segura et al. (specific signatures for RS, OIS, IRIS), and the age‐associated human genes from the GTEx database tissues (Dong et al., 2021), we found that nine subtelomeres (chr1p, chr8q, chr9q, chr11p, chr16p, chr16q, chr19p, chr20q, and chr22q) are significantly enriched in age/senescence‐related upregulated genes (Figure S5). Then, we analyzed the connections between chromosome ends and the different senescence inducers described above in addition to the OIS transcriptome published in (Martínez‐Zamudio et al., 2020). This was performed by calculating the proportion of upregulated DEGs in any given subtelomere as compared to the DEGs found in all subtelomeres (Figure 3b). As expected, the nine subtelomeres enriched for upregulated DEGs were clustered (Figure 3b, the purple and the yellow cluster) together with other two chromosome ends, 17q and 19q. Interestingly, the type of inducer (RS or OIS) also clustered indicating inducer‐specific effects on the choice of the subtelomere preferentially upregulated (Figure 3b). Noteworthy, even if the IRIS DEGs did not exhibit any significant enrichment in specific subtelomeres, they show the same localization trend toward the subtelomeres enriched for the other inducers (Figure 3b).

### 2.5 Subtelomeres with upregulated DEGs are enriched in factors controlling insulation, cohesion and polycomb repression

**FIGURE 4:**
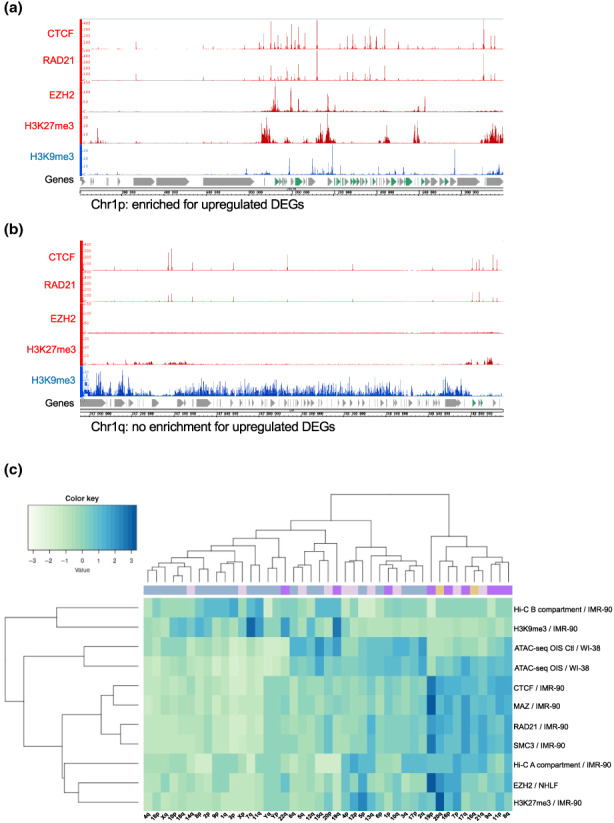
Histone mark and transcription factor enrichment distribution at subelomeres. (a) Example of a chromosome end with enrichment and (b) no enrichment for upregulated DEGs displaying different ChIP‐seq factors from ENCODE. Factors with positive (red) and negative (blue) enrichment in IMR‐90 primary fibroblasts are shown. Upregulated DEGs are marked in green. (c) Heatmap showing the clustering of different histone marks and other factors for the 2 Mb of the telomere end. The color line at the top of the heatmap correspond to the clusters identified in Figure 3b.

We then investigated whether particular features are characteristic of the nine subtelomeres described above. For that, we used publicly available ChIP‐seq data of histone marks and transcription factors coming from proliferative primary fibroblasts and examined the coverage of such factors across the entire 2 Mb subtelomeric region. The coverage was computed as a percentage, and Spearman's rank correlation coefficient was calculated by comparing the nine subtelomere ends with upregulated DEGs vs the rest of the subtelomeres (Table S2). We found that enriched chromosome ends with upregulated DEGs have a positive correlation with the CCCTC‐binding factor (CTCF) and the Myc‐associated zinc finger protein (MAZ). Interestingly, these factors, together with members of the cohesin complex such as RAD21 and SMC3, which were also highly correlated with these chromosome ends, are associated with insulation and chromatin looping (Table S2 and Figure 4a,b).

Besides, we found a correlation of several histone marks such as H3K27me3, H4K20me1, H3K36me3, H3K4me3, H3K79me2, and H3K9ac. Amongst those, the polycomb H3K27me3 factor appeared to be an important regulatory element of upregulated DEG subtelomeres as also the histone methyltransferase enzyme Enhancer of Zeste Homolog 2 (EZH2), which catalyzes the addition of methyl groups to histone H3 at lysine 27, was present in our correlation analysis (Table S2 and Figure 4a,b). We also found a negative correlation of marks such as H3K9me2 or H3K9me3, suggesting the chromosome ends with positive enriched DEGs are preferentially associated with facultative rather than constitutive heterochromatin. Overall, the subtelomeres can be partitioned into H3K27me3‐cohesin‐CTCF enriched ends containing genes susceptible of being upregulated at senescence and H3K9me3 enriched ends that appear resistant to senescence‐associated derepression.

Next, we extended our analysis by generating heatmaps (Figure 4c) using different factors enriched in our correlation analysis (Table S2) coming primarily from proliferative IMR‐90 primary fibroblast ChIP‐seq data from ENCODE. In addition, we used ATAC‐seq datasets from OIS cells to unveil chromatin accessibility (Martínez‐Zamudio et al., 2020) and Hi‐C data (Rao et al., 2014) which reveals long‐range interaction in the genome describing two types of chromatin compartments, one associated with open chromatin called A‐type and the other associated with closedaut chromatin or B‐type (Lieberman‐Aiden et al., 2009).

Heatmaps were made by using the coordinates of all ChIP‐seq peaks, ATAC‐seq and Hi‐C data to calculate the length they cover across each subtelomere followed by standardization of the data (Figure 4c). We found that eight out of the 11 chromosome ends of the purple and yellow cluster enriched in upregulated DEGs (see Figure 3b) are clustered with the A‐type chromatin compartment and exhibit a high level of CTCF, MAZ, RAD21, SMC3, EZH2 factors, and H3K27me3. In contrast, the three other subtelomeres enriched in upregulated DEGs (1p, 19q, and 22q) clustered with H3K9me3 and the B‐type compartment.

**FIGURE 5:**
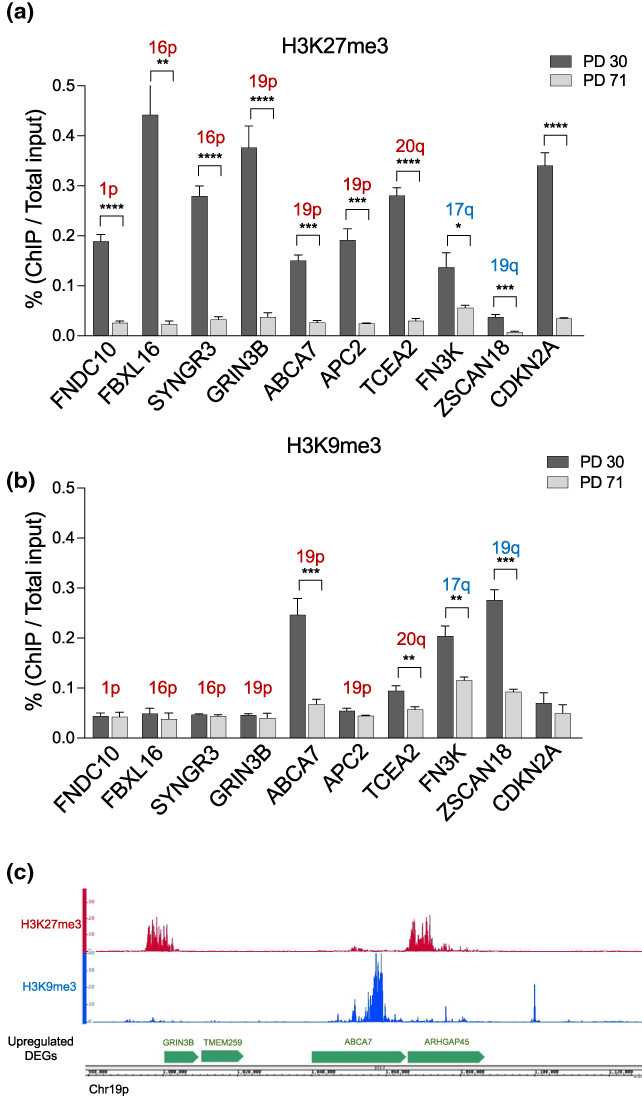
ChIP‐qPCR of upregulated DEGs. ChIP performed with H3K27me3 (a) H3K9me3 (b) antibodies in young (PD 30) and fully senescent MRC‐5 cells (PD 71). Upregulated DEGs found at positively enriched subtelomeres are marked in red, while in blue are marked subtelomeres containing upregulated DEGs but not positively enriched. CDKN2A was used as a positive control for H3K27me3 enrichment and is not located at a subtelomeric site. The bars represent the mean ± SD of three biological replicates. Statistics were performed using a *t*‐test, *p < 0.05; **p < 0.001; ***p < 0.0001; ****p < 0.00001. (c) Example of read profiles of H3K9me3 and H3K27me3 ChIP‐seq obtained from IMR‐90 ENCODE data in a fragment of a subtelomeric region of chromosome 19p.

Finally, we performed H3K27me3 and H3K9me3 ChIP‐qPCR in MRC‐5 cells at PD 30 and PD 71 (senescent cells) to test the enrichment in a selection of upregulated DEGs (Figure 5). The genes tested were mainly found in subtelomeres significantly enriched for upregulated DEGs (Figure 5a and Figure S6) and highly correlated with H3K27me3 histone mark (chr1p, chr16p, chr19p and chr20q). In agreement with ENCODE data, we found that those DEGs were enriched in H3K27me3 in young cells and as predicted, the levels significantly decrease in senescent MRC‐5 cells. We further tested three genes predicted to be enriched on H3K9me3 by ENCODE, and again, we confirmed those results (Figure 5b). One of those genes, *ABCA7*, was enriched on H3K9me3 and flanked by two genes enriched on H3K27me3 (Figure 5c), suggesting a complex regulation of gene expression at senescence.

## 3 DISCUSSION

Here, we revealed specific chromatin features of human subtelomeres contributing to the transcriptome of senescent and aged cells. Using the MRC‐5 cell line, we found enrichment of upregulated genes in a subset of subtelomeres (defined as the chromosomal regions up to 2 Mb from the telomere). These upregulated genes are likely to be normally repressed in young cells since they are specifically enriched in subtelomeres and are less expressed than the other subtelomeric genes in young cells. A similar subtelomere enrichment is found using public datasets of RS and OIS but not in IRIS. Using clustering analyses, our data confirmed that most upregulated DEGs are associated with a small number of subtelomeres even in the case of IRIS.

We further showed that the derepression of some of these genes is dependent upon the decreased levels of TRF2 and Lamin B1 occurring at senescence. Moreover, knocking down *TERF2* and *LMNB1* genes in young cells is sufficient to derepress their expression. Since TRF2 is known to mediate the formation of chromatin loops between telomeres and subtelomeres by the interaction with interstitial telomeric sequences (ITS; Kim et al., 2016; Robin et al., 2014, 2020), it is likely that the higher‐order organization of subtelomeres controlled by TRF2 plays a role in the senescence‐associated gene derepression observed at subtelomeres. One cannot rule out that other telomere factors, potentially able to interact with ITS, such as TRF1 (Simonet et al., 2011) or TERRA (Feretzaki et al., 2020), could mediate chromatin loops affecting subtelomeric gene expression. Indeed, TERRA favors the formation of heterochromatin marks by binding ORC1 (Deng et al., 2009) and the EZH2 and SUZ12 polycomb factors (Montero et al., 2018).

These results raised the question of whether this subset of subtelomeres has peculiar chromatin composition. Indeed, as a result of a large screening of ChIP‐seq data from primary young fibroblasts, we found that these subtelomeres are specifically enriched with proteins such as CTCF, MAZ, cohesin (RAD21, SMC3), and polycomb factors (EZH2, H3K27me3). Remarkably, CTCF, MAZ, and the cohesin complex are important for the formation of chromatin loops (Rao et al., 2017; Xiao et al., 2021). It is also known that cohesin and polycomb proteins interact to maintain chromatin organization (Dorsett & Kassis, 2014; Schaaf et al., 2013). Moreover, the expression of many of those factors decreases upon senescence (Ito et al., 2018).

Clustering analyses showed that the subtelomeres with a high proportion of upregulated DEGs are preferentially associated with the A‐type chromatin compartment and become more open upon OIS induction. One limitation of this analysis is the heterogeneity of cell lines and in most ChIP‐seq cases the lack of corresponding RNA‐seq data. Nevertheless, our analyses unveil that a subset of subtelomeres has specific chromatin features associated with gene upregulation at senescence which could render them more susceptible to gene expression at senescence.

All this raises the question of a role of telomere DNA length in the regulation of subtelomeric gene transcription during replicative senescence. Previously, the expression of the subtelomeric gene, ISG15, has been directly associated with telomere length (Lou et al., 2009). However, we did not find ISG15 in our RNA‐seq results nor in the published transcriptome dataset of replicative senescence we used in our analyses. Arguing against a direct role of telomere length is the fact that we also found an enrichment of upregulated subtelomeric genes in OIS, a situation where the average telomere length is unaltered (Suram et al., 2012). However, in the case of OIS, one cannot rule out a telomere effect on subtelomeric gene expression caused by DNA replication stress leading to stochastic telomere loss, which are telomere events that cannot be detected by measuring the mean telomere length (Suram et al., 2012). Other non‐exclusive possibilities are an alteration at senescence of telomere‐subtelomeric chromatin loops mediated by factors such as TRF2 (Kim et al., 2016; Robin et al., 2014, 2020), the expression of which is known to decrease upon p53 activation (Fujita et al., 2010; Mendez‐Bermudez et al., 2022) or a subtelomeric enrichment of Polycomb‐repressed genes, as shown in this work, being up‐regulated as a consequence of the general attenuation of polycomb repression occurring during senescence (Bracken et al., 2007; Jacobs et al., 1999).

Altogether, these results unveil the existence of a subset of subtelomeres with specific chromatin profiles enriched in genes prone to be derepressed during senescence. This highlights the importance of subtelomeric regions in the senescence process.

## 4 MATERIALS AND METHODS

### 4.1 Cell lines

MRC‐5 human primary lung fibroblasts were obtained from ATCC. MRC‐5 cells were grown in DMEM supplemented with 10% fetal bovine serum, penicillin (100 IU/mL) and streptomycin (100 μg/mL). Cells were cultured at 37°C, 5% CO_2_ and 5% O_2_.

### 4.2 Lentivirus infection and siRNA transfection

Lentiviral particles were produced with 293 T cells transiently transfected with the virus packaging plasmids p8.91, VSVg and the lentiviral expression vector (empty: pWPIR‐GFP or TRF2: pWPIR‐GFP‐TRF2) by calcium phosphate precipitation. Viral supernatants were collected 48 h after transfection and concentrated by overnight centrifugation at 4°C.

siRNAs (Control and LMNB1) were purchased from Dharmacon (On‐Target Plus SMARTpool) and transfected with DharmaFECT1 transfection reagent (Dharmacon) for 3 days. The efficiency of siRNA was assessed by RT‐qPCR.

### 4.3 RNA extraction and RT‐qPCR

RNA extractions were performed accordingly to the manufacturer's instruction using the RNeasy Plus Mini Kit (Qiagen). The concentration and quality of the extracted RNA were assayed using the Nanodrop 2000 (Thermo Scientific) and/or Bioanalyzer instrument (Agilent). One μg of RNA was reverse‐transcribed using High‐Capacity RNA‐to‐cDNA Kit (Thermo Fisher Scientific) in a T100 thermal cycler (Bio‐Rad). Quantitative PCR was performed using 10 ng of cDNA with 0.6 μM primers (see list below) and SYBR Green Master Mix (Roche) on a StepOne plus thermocycler (Applied Biosystems). Data were analyzed using the Pfaffl method after the calculation of primer efficiency. VAMP7 and GAPDH were used as endogenous controls. All reactions were performed in triplicates and at least three biological replicates were used to generate each dataset.

**Table:** 

Gene name	Forward primer	Reverse primer
*PTGDS*	CTCTACAGCCGAACCCAGAC	CAGAGACATCCAGAGCGTGG
*ABCA2*	AAGAACGTGACGCTCAAACG	AAGGAGACTTCCTTCACGGAG
*NPDC1*	CTGGACTGTGCCCTGAAGAG	TGGGCCAGGAAGTCAATCTC
*SAPCD2*	ATGCTGAAGGAGCAGAACCG	GCTTAATGAGCGCCGACTTC
*DPP7*	CTGGTGTCGGACAGGTTCTG	GACTTCCCGTAGTAGCGGTG
*RNF208*	TGGCTGTCAACACGTCCATC	TACTACATGATGGAGCAGGCG
*CYSRT1*	GAGGACAGACTGCCGTGTTG	GCATCTCCGAGGATGAGGAAC
*PNPLA7*	GTACCCACAGGTGGTGACTC	CTTCCTCTGACACGGGCATC
*LMNB1*	ACATGGAAATCAGTGCTTACAGG	GGGATACTGTCACACGGGA
*TERF2*	GTTGGAGGATTCCGTAGCTG	GACCTTCCAGCAGAAGATGCT
*VAMP7*	GCCCATCCTGTTATCCAGAA	CAAAGCCTTTTTGGCCATC
*GAPDH*	TTGCCATGTGAGTACGTTAGT	CCGGACAGACTGAAGCCAT

### 4.4 EdU proliferation assay

To measure the proliferation of MRC‐5 cells, the click‐iT EdU Alexa Fluor 647 Imaging Kit (Thermo Fisher Scientific) was used. EdU was incubated for 14 h at a final concentration of 10 μM. Cells were imaged using fluorescence microscopy.

### 4.5 Chromatin immunoprecipitation (ChIP)

Around 5 million cells were cross‐linked for 10 min with 1% formaldehyde and washed with cold PBS. The cells were centrifuged, and the pellet re‐suspended in cell lysis buffer (5 mM PIPES pH 8, 85 mM KCl, 0.5% NP40 and protease inhibitors). The cells were pelleted at 4°C and re‐suspended in nucleus lysis buffer (50 mM Tris pH 8, 10 mM EDTA, 1% SDS, protease inhibitors), and the cells were sonicated using a Bioruptor Pico (Diagenode) to get an average fragment size of 600 bp. Each ChIP was carried out with 25 μg of DNA and incubated overnight with the desired antibody. Magnetic beads (Dynabeads, Life Technologies) were added for 2 h. The beads were washed with a low salt buffer (150 mM NaCl, 1% Triton X‐100, 0.1% SDS) and a high salt buffer (500 mM NaCl, 1% Triton X‐100, 0.1% SDS), followed by a lithium salt buffer (0.25 M LiCl, 1% NP40, 1% deoxycholic acid). Chromatin was eluted with a 1% SDS, 0.1 M NaHCO3 solution, and the cross‐link was reversed at 65°C overnight. The DNA was treated with RNase (10 mg/mL for 20 min) and proteinase K (10 mg/mL for 1 h at 55°C) followed by phenol‐chloroform purification and ethanol precipitation.

### 4.6 RNA sequencing

Young MRC‐5 cells were infected with an empty (pWPIR‐GFP) or TRF2 expressing (pWPIR‐GFP‐TRF2) virus at a multiplicity of infection (MOI) of one. Two days after infection, cells were transfected with a siRNA control or siLMNB1 and collected for RNA extraction 4 days after, at PD 30. For senescent cells, MRC‐5 cells were transduced with a TRF2‐expressing vector for about 25 PD before they senesce. Total RNA was extracted at the senescent point (for control cells at PD 71 and PD 75 for TRF2‐expressing cells). Each condition was done in triplicates. The proliferative and senescence status of cells were assessed constantly with EdU proliferation assay and Senescence‐Associated *β*‐Galactosidase staining.

Paired‐end sequencing (read length: 2 × 150 bp) was performed by NovoGene using an Illumina sequencer. Between 30 and 50 million reads were obtained per sample. Raw reads were trimmed using Trimmomatic (v0.39) with a minimal read length of 35 bp. Trimmed reads were mapped to the human genome assembly (GRCh38) using STAR (v2.6.1d) with the ‐quantMode GeneCounts (Dobin et al., 2013). Differentially expressed genes (DEGs) analysis was performed using DESeq2 R package (Love et al., 2014). *p*‐Values were corrected for multiple testing using the Benjamini and Hochberg default method. The list of differentially expressed genes was obtained with a cutoff of 0.05 on adjusted p‐values. A log2 fold‐change value of 1 (for DEG analysis between senescent and young MRC‐5 cells) and 0.58 (for DEG analysis between young MRC‐5 transfected with siLMNB1 and siRNA control) was used. Protein coding genes and pseudogenes were kept for subsequent analysis.

### 4.7 Senescence‐associated *β*‐galactosidase (SA‐β‐Gal) assay

SA‐*β*‐Gal staining was performed using the Senescence Detection Kit (ab65351; Abcam) according to the manufacturer's instructions. Cells were visualized using phase‐contrast microscopy and the percentage of SA‐*β*‐Gal positive cells was calculated.

### 4.8 Statistical analysis

Statistical analysis was performed using the GraphPad Prism software v9. Student's *t*‐test and the corresponding nonparametric Mann–Whitney test were used for qPCR analysis. The Kruskal–Wallis test was used for DESeq2 counts analysis. Differences were considered statistically significant when the *p*‐value was <0.05.

The p‐value of the enrichment analysis was based on the cumulative distribution function of the hypergeometric distribution using the function *phyper* from the stats R package. Fold enrichment (FE) was calculated using the following equation:

$$\mathrm{FE}=\frac{k}{\frac{s*M}{N}}$$

where *k* represents the number of successes, *s* is the sample size, *M* is the number of successes in the population, and *N* is the size of the population.

Regarding the enrichment analysis in the distribution of the DEGs in subtelomeres, the distance to the closest telomere was calculated for each gene. Chromosomes were then divided into 2 Mb non‐overlapping intervals according to the distance from a telomere, and DEGs were grouped in those intervals. The enrichment analysis was calculated as described above, with *s* as the total number of genes of interest, *M* as the total number of genes in the interval, and *N* as the total number of genes in the genome.

The correlation analysis between subtelomere ends with upregulated DEGs vs the rest of subtelomeres and different ENCODE factors was performed by calculating the length of the overlap between the coordinates of DEGs and the ones of the ChIP‐seq peaks. This overlap was expressed as a percentage followed by a Spearman's rank correlation coefficient computed using the R package lares.

A cluster heatmap (Figure 3b) was generated by calculating the percentage of upregulated genes present in the different chromosome ends. For each data set, the percentage was calculated as the number of upregulated genes in one chromosome end divided by the total number of upregulated genes in all chromosome ends. Heatmap of C was generated using the coordinates of different ChIP‐seq peaks from a selection of young primary fibroblast available in ENCODE, ATAC‐seq and Hi‐C data. The coordinates were used to calculate the length they covered across each subtelomere and then standardized using the *scale* function in R.

## AUTHOR CONTRIBUTIONS

Methodology, Martin Rey‐Millet; Mélanie Pousse; Chan Soithong; Aaron Mendez‐Bermudez Software, Mélanie Pousse; Chan Soithong Formal Analysis, Martin Rey‐Millet; Mélanie Pousse; Jing Ye; Aaron Mendez‐Bermudez; Eric Gilson Conceptualization, Aaron Mendez‐Bermudez, Eric Gilson; Writing‐Original Draft, Martin Rey‐Millet; Aaron Mendez‐Bermudez, Eric Gilson; Funding Acquisition, Eric Gilson.

## CONFLICT OF INTEREST STATEMENT

The authors declare no competing interests.

## Supporting information


Figure S1

Table S1

Table S2

## Data Availability

RNA‐seq data were deposited in the Gene Expression Omnibus database under accession numbers GSE180406 and GSE160503.
