# Supplementary material for: Senescence‐associated transcriptional derepression in subtelomeres is determined in a chromosome‐end‐specific manner
Source: Aging Cell. 2023 Mar 15;22(5):e13804. doi: 10.1111/acel.13804 (PMC10186611; doi:10.1111/acel.13804)
Supplement: Supplementary file 1 — Figure S1 [file ACEL-22-e13804-s002.pdf]

Figure S1 related to Figure 1

A

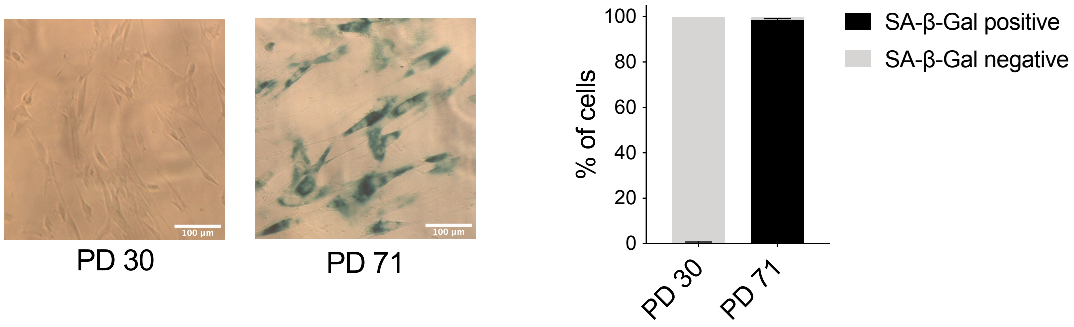


B

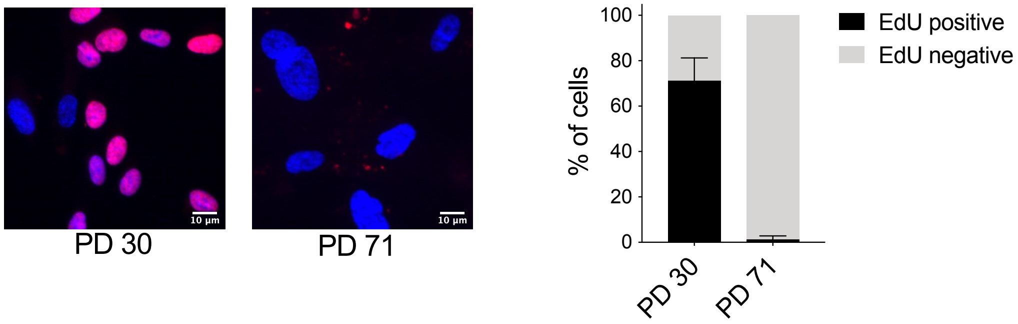


**Figure S1. Characterization of senescent cells**
(A) Young (PD 30) and senescent (PD 71) MRC-5 cells assayed for Senescence-Associated $\beta$-Galactosidase (SA-$\beta$-Gal) and (B) EdU incorporation for 14 h at 10 µM concentration. Error bars show the standard deviation of n = 3.

Figure S2 related to Figure 1

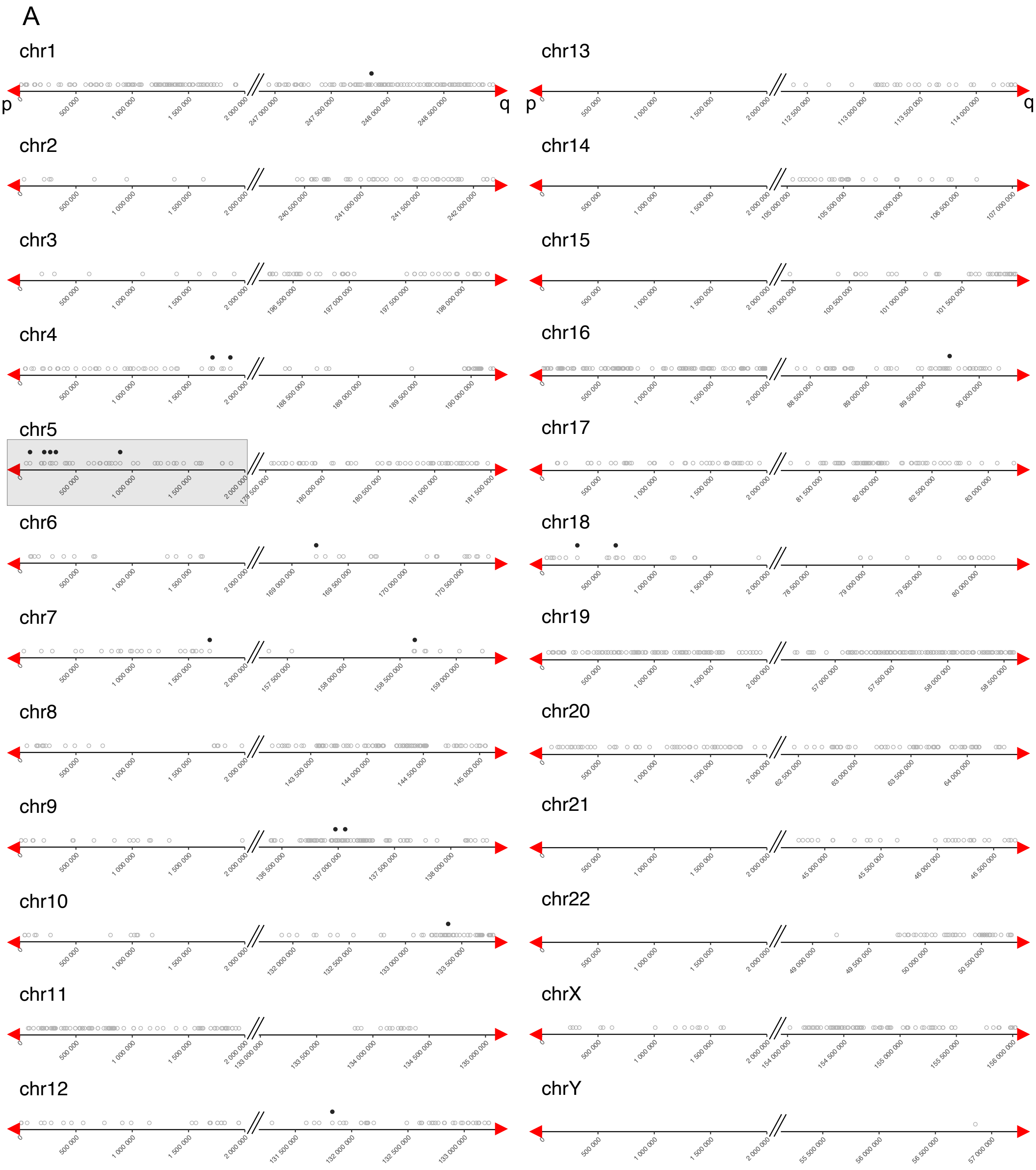


**Figure S2. Distribution of downregulated genes at the first 2 Mb from telomeres**
(A) Downregulated DEGs in the first 2 Mb from telomeres are depicted. Each dot represents a gene while red arrows mark the start of the telomere. All protein-coding genes and pseudogenes are represented by grey empty dots. Black dots represent downregulated genes (senescent versus young MRC-5 cells) from this work. Grey boxes delimitate the subtelomeres that are significantly enriched in downregulated DEGs.

# Figure S3 related to Figure 2

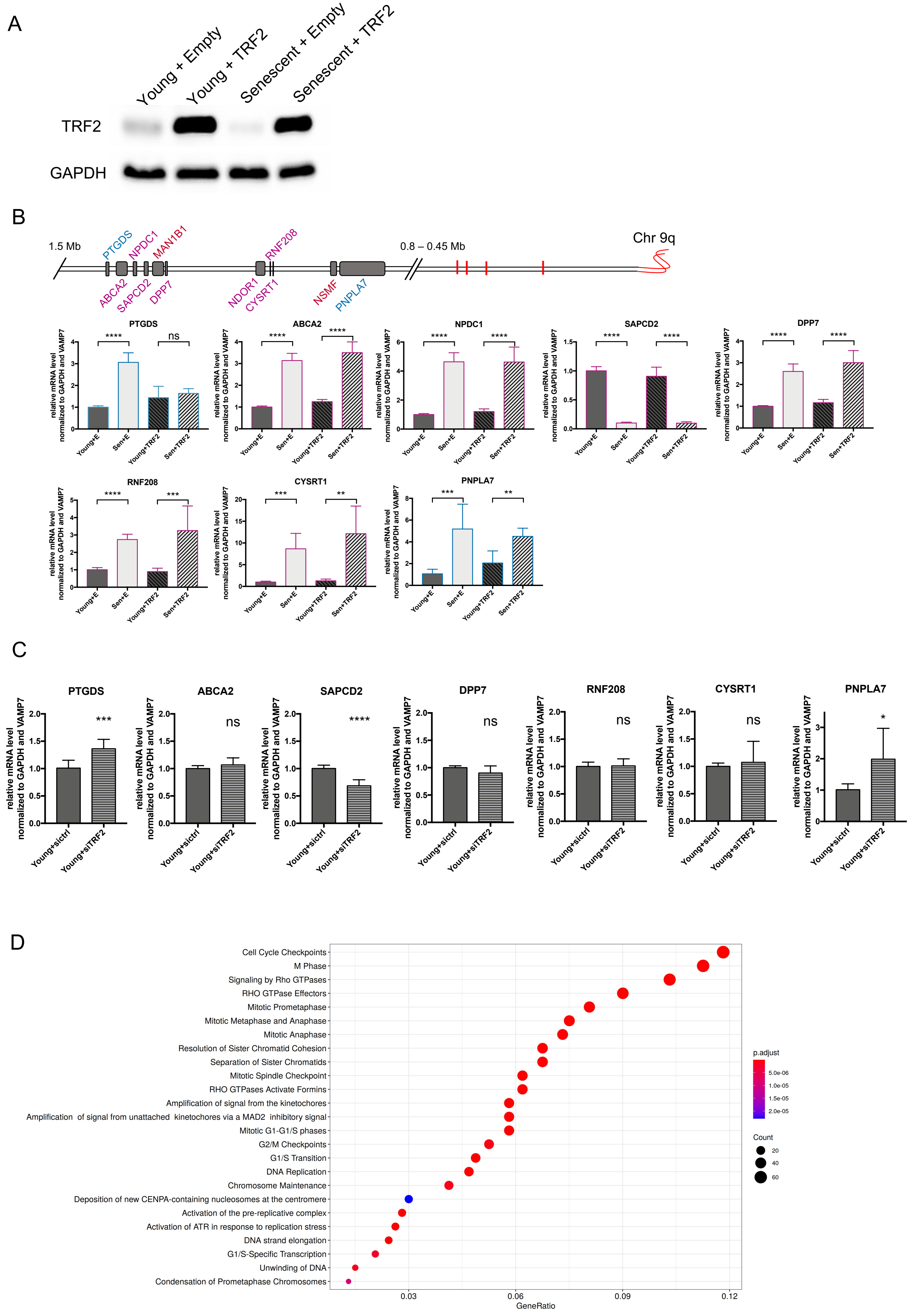

**Figure S3. TRF2 protein levels impact subtelomeric gene expression**

(A) Western blotting of young and senescent MRC-5 cells transduced with an empty or a TRF2-expressing vector. Young cells were transduced for 6 days while senescent cells were transduced 25 population doublings before they senesce. (B) Schematic representation of chromosome 9q subtelomere. Expressed genes found by RNA-seq are shown by a grey rectangle. The name of the gene is differentiated by $TRF2^{low}$-dependent (blue color), $TRF2^{high}$-dependent (red color), and TRF2-independent (purple color). Interstitial telomeric sequences are represented by vertical red bars. Gene expression by RT-qPCR in young and senescent (MRC-5, infected with an empty (E) or a TRF2-expressing vector (TRF2), normalized to two housekeeping genes (GAPDH and VAMP7) are shown. Bars represent the mean ± SD of three biological replicates. Statistics were performed using a t-test or Mann-Whitney test, $^{**}P < 0.001$; $^{***}P < 0.0001$; $^{****}P < 0.00001$. (C) Reactome pathway analysis of DEG defined as TRF2-independent gene category described in Figure 2A. (D) Gene expression by RT-qPCR in young cells with TRF2 knockdown for 72 h. Mean ± SD of three biological replicates is shown.

Figure S4 related to Figure 2

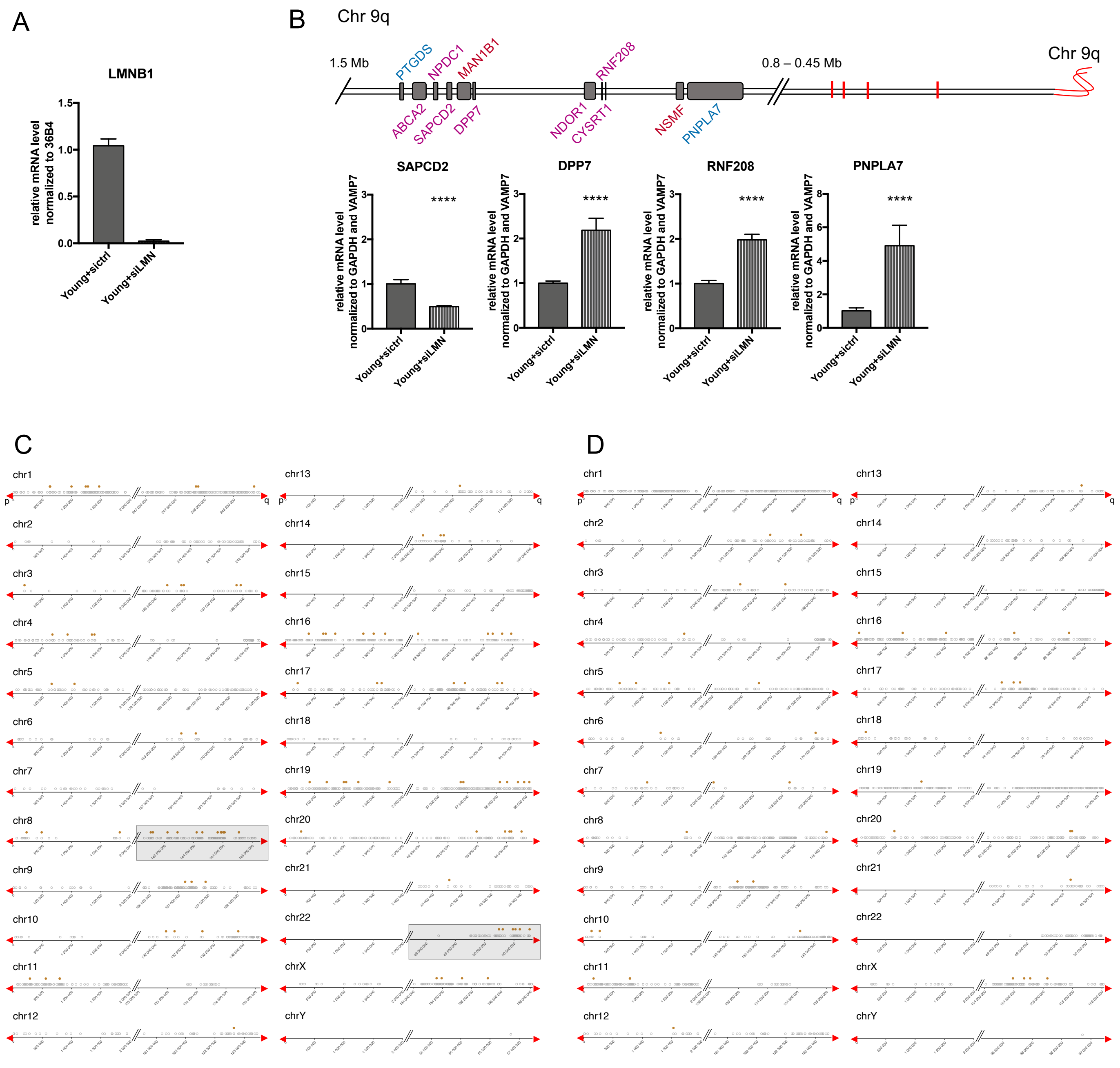


**Figure S4. Lamin B1 downregulation influences subtelomeric gene expression**

(A) Verification of LMNB1 expression in young MRC-5 after 3 days incubation of siLMNB1 or control (sictrl). (B) Schematic representation of chromosome 9q subtelomere and expression of some subtelomeric genes by RT-qPCR in young (PD 30) MRC-5 transfected with a siRNA control or siLMNB1, normalized to two housekeeping genes (GAPDH and VAMP7). Data show the mean ± SD of three biological replicates. Statistics were performed using either t-test or Mann-Whitney test; **** $P < 0.0001$. (C) Visualization of upregulated and (D) downregulated genes in young MRC-5 cells transfected with siLMNB1 versus controls in the first 2 Mb from all telomeres (brown dots). Each dot represents a gene and is plotted on the x-axis according to its location (length in base pairs). Grey empty dots show all protein-coding genes and pseudogenes. Grey boxes delimitate the subtelomeres that are significantly enriched in DEG.

Figure S5 related to Figure 3

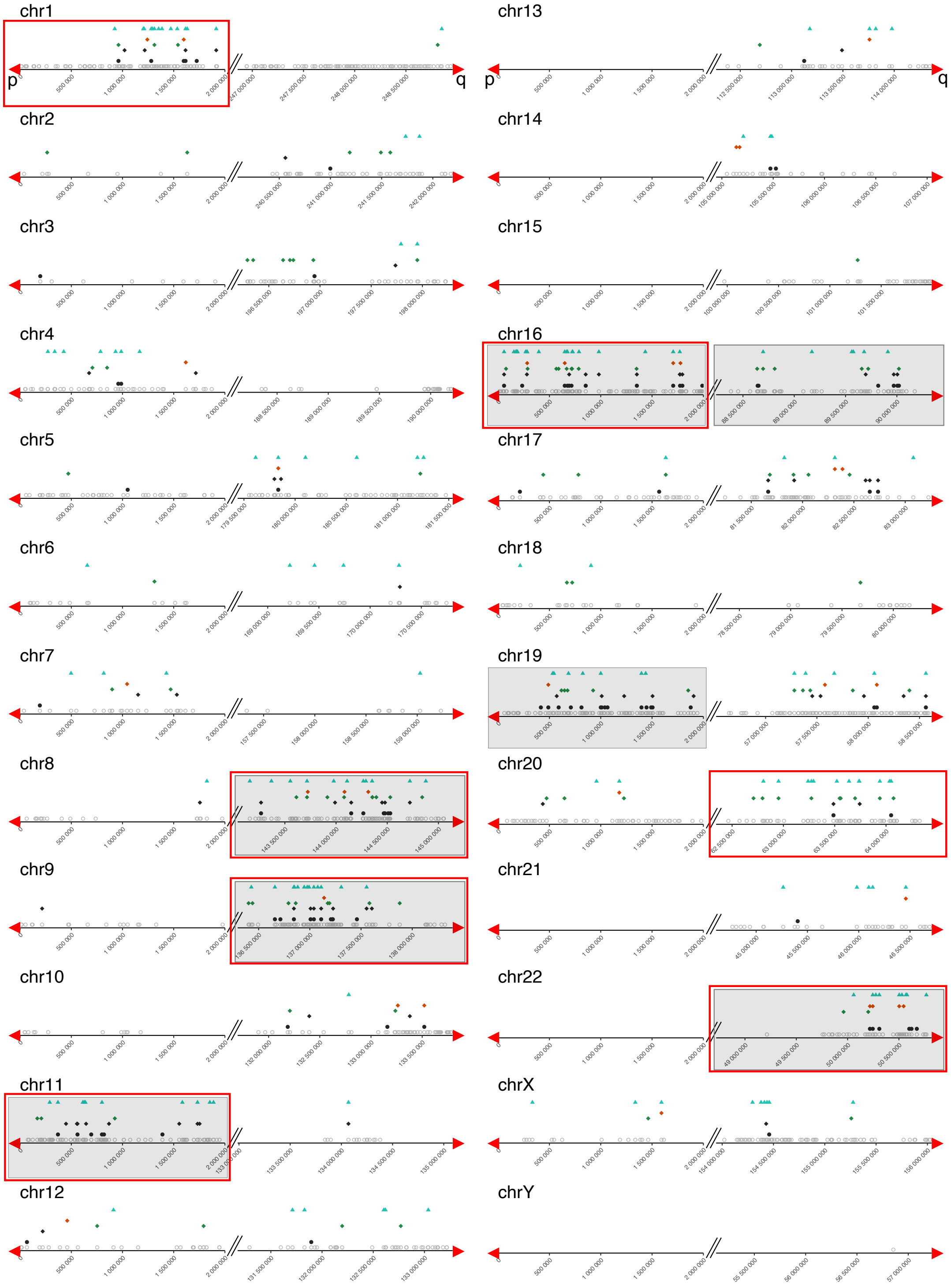


**Figure S5. Subtelomeric DEG distribution of different senescence inducers** Upregulated genes in the first 2 Mb from telomeres are depicted. Each dot represents a gene while red arrows mark the start of the telomere. All protein-coding genes and pseudogenes are represented by grey empty dots. Black dots represent upregulated genes (senescent versus young MRC-5 cells) from this work. Black diamonds depict upregulated genes in replicative senescence identified in published datasets (Hernandez-Segura et al., 2017), green and red diamonds are upregulated genes found in oncogene-induced senescent and ionizing radiation-induced senescence respectively (Hernandez-Segura et al., 2017). The turquoise triangles correspond to upregulated genes in aged versus young tissues (Dong et al., 2021). Grey boxes delimitate the subtelomeres that are significantly enriched in upregulated DEGs (senescent vs young) from this work while red frames delimitate the subtelomeres significantly enriched in genes upregulated with age shown by Dong *et al*., 2021.

Figure S6 related to Figure 5

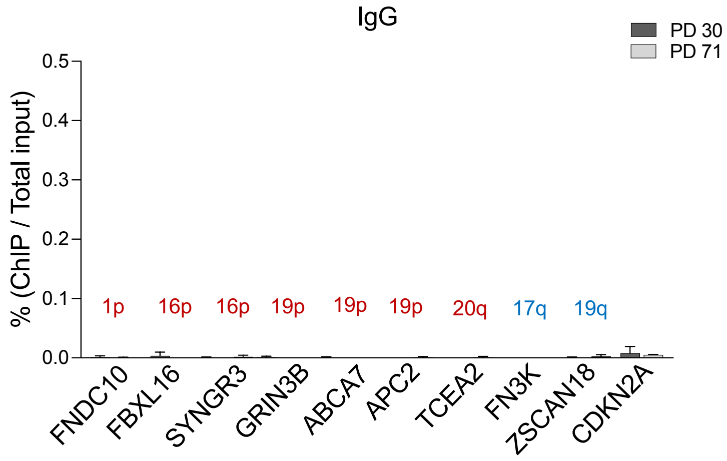


**Figure S6. ChIP-qPCR of upregulated DEGs**

IgG control for ChIP-qPCR experiments shown in Figure 5.
