# Supplementary material for: Senescence‐associated transcriptional derepression in subtelomeres is determined in a chromosome‐end‐specific manner
Source: Aging Cell. 2023 Mar 15;22(5):e13804. doi: 10.1111/acel.13804 (PMC10186611; doi:10.1111/acel.13804)
Supplement: Supplementary file 2 — Table S1 [file ACEL-22-e13804-s001.pdf]

	1p	3q	8q	9q	11p	16p	16q	19p	20q	22q
RS vs young [a]			1.91e-4	4.40e-7	7.69e-4	6.09e-8	6.22e-5	2.21e-11		3.71e-5
siLMNB1 vs siCtrl [b]			6.73e-5							6.07e-3
RS signature [c]				2.92e-3	2.63e-4	7.25e-4				
OIS signature [c]		6.11e-4	2.20e-3	9.60e-3			5.94e-3		1.85e-4	
IRIS signature [c]										1.34e-3
GTEx dataset [d]	8.74e-4		3.80e-3	4.98e-4	5.86e-3	8.87e-7			4.69e-5	3.76e-4

Supplementary table 1. Subtelomere enrichment for upregulated DEGs of different senescence inducers

Summary of chromosome ends obtained by enrichment analysis of upregulated DEGs upon different senescent stimuli. [a] Replicative senescence (RS) vs young MRC-5 cells and [b] siLMNB1 vs siControl in young cells from this work; RS, [c] oncogene-induced senescence (OIS), ionizing radiation-induced senescence (IRIS) from Hernandez-Segura *et al*. and from [d] aged tissues from the GTEx database from Dong *et al*. The *p*-value of the enrichment was obtained using a hypergeometric test.
