# Supplementary material for: Senescence‐associated transcriptional derepression in subtelomeres is determined in a chromosome‐end‐specific manner
Source: Aging Cell. 2023 Mar 15;22(5):e13804. doi: 10.1111/acel.13804 (PMC10186611; doi:10.1111/acel.13804)
Supplement: Supplementary file 3 — Table S2 [file ACEL-22-e13804-s003.pdf]

Factor_Cell line	Correlation value	*p* value
	**Positive correlation**	
CTCF_HFFc6	0.516572	6.7636032902782e-07
MAZ_IMR90	0.515417	7.23256950583623e-07
CTCF_BJ	0.514085	7.81140356708368e-07
CTCF_IMR90	0.511593	9.01416044558095e-07
CTCF_AG10803	0.487943	3.32108640069207e-06
CTCF_AG04450	0.487943	3.32108640069207e-06
CTCF_AG04449	0.485453	3.78853290070119e-06
CTCF_AG09319	0.482964	4.3173305066657e-06
CTCF_WI38	0.482365	4.45447632047301e-06
CTCF_HFFMyc	0.47799	5.58808132588906e-06
H3K27me3_SJCRH30	0.474454	6.69675457725983e-06
H4K20me1_IMR90	0.465626	1.04309845399865e-05
USF2_IMR90	0.463771	1.14311769264503e-05
H3K36me3_BJ	0.453228	1.90451726393911e-05
CTCF_AG09309	0.451237	2.09326854662105e-05
H3K4me3_BJ	0.45061	2.15628162896894e-05
H3K4me3_AG04449	0.449365	2.28659939853691e-05
H3K4me3_AG09309	0.44936	2.28712563038703e-05
H3K4me3_HFFMyc	0.44069	3.42031131813033e-05
SMC3_IMR90	0.440657	3.42557377270612e-05
H3K4me3_AG04450	0.440652	3.42632606911855e-05
EZH2_IMR90	0.439383	3.6307674386682e-05
H3K4me3_AG09319	0.433176	4.80559002628514e-05
MXI1_IMR90	0.430778	5.34750663907591e-05
H3K4me3_SJCRH30	0.429439	5.67416116126498e-05
H3K4me3_AG10803	0.424463	7.05830384270416e-05
H3K4me3_WI38	0.423218	7.45042113453182e-05
RAD21_IMR90	0.416994	9.73234774374079e-05
H3K79me2_IMR90	0.413336	0.000113596087707541
H3K4me3_GM23248	0.4033	0.000172063639269657
H3K9ac_IMR90	0.402099	0.000180670571791162
	**Negative correlation**	
H3K9me2_GM23248	-0.272429	0.0132823945358119
MAFK_IMR90	-0.268871	0.0145855503472771
H3K9me3_IMR90	-0.261398	0.0176862409571468
H3K9me3_GM23248	-0.220121	0.0469082004645685

Supplementary table 2. Correlation coefficient between nine enriched subtelomeres with upregulated DEGs vs the rest of subtelomeres.
